# Comparing delayed versus on-arrival administration of a modified live viral vaccine in feedlot cattle

**DOI:** 10.18849/ve.v7i2.502

**Published:** 2022-06-15

**Authors:** Ashlee Ambs+, Heather Moberly+, Sarah Capik+

**Keywords:** BOVINE RESPIRATORY DISEASE, CATTLE, FEEDLOT, MODIFIED LIVE VIRUS, MORBIDITY, VACCINE TIMING

## Abstract

PICO question

In auction market calves at high risk of developing bovine respiratory disease (BRD), does delayed (14–30 days) vaccination with a modified live vaccine (MLV) for viral respiratory pathogens versus administration of MLV on-arrival (within 24 hours of arrival) to the feedlot, result in a decreased percentage of calves with BRD morbidity diagnosed based on visual signs and rectal temperature >40 degrees Celsius?

Clinical bottom line

Category of research question

Treatment

The number and type of study designs reviewed

Two papers were critically reviewed. Both are randomised complete block designs

Strength of evidence

Weak

Outcomes reported

Delaying administration of a modified live respiratory vaccine to feedlot cattle may result in lower BRD retreatments

Conclusion

In feedlot calves, delaying modified live vaccine administration for viral respiratory pathogens may result in lower BRD retreatment rates than cattle receiving the vaccine on arrival to the feedlot. Significant statistical data from one study supported this conclusion while another showed numerically less retreatments in calves vaccinated on arrival versus delayed vaccination

How to apply this evidence in practice

The application of evidence into practice should take into account multiple factors, not limited to: individual clinical expertise, patient’s circumstances and owners’ values, country, location or clinic where you work, the individual case in front of you, the availability of therapies and resources.

Knowledge Summaries are a resource to help reinforce or inform decision making. They do not override the responsibility or judgement of the practitioner to do what is best for the animal in their care.

## Clinical scenario

It is common practice in North American feedlots to use a multivalent modified live vaccine (MLV) for viral respiratory pathogens during arrival processing as a management strategy to reduce bovine respiratory disease (BRD) risk. However, BRD continues to be a financial and health issue in feedlot cattle despite consistent use of on-arrival MLV for viral respiratory pathogens. Recently, modifications to protocols, such as altering vaccine timing, have been considered in the hopes of improving vaccine efficacy and reducing BRD morbidity. Changing vaccine timing would also allow cattle to acclimate to their new environment before challenging them with a vaccine. Feedlot producers and veterinarians must decide on the timing of vaccine administration by reviewing research evidence and considering the financial and production impacts of those decisions.

## The evidence

The strongest evidence for research is presented in systematic reviews and several were identified in our search. However, they did not answer the specifics of the presented question and thus were excluded from the review. The two studies reviewed in this summary are randomised controlled trials*, which produce the strongest evidence of experimental studies (Hagenmaier et al., 2018; and Rogers et al., 2016). Though each of these studies evaluated a large number of cattle, the experimental unit for each study was pen which means the sample sizes for detecting differences in each study are relatively small. Small randomised controlled trials have a lower estimate of the true outcome of the treatments if applied outside of the study compared to large randomised controlled trials (O’Connor & Fajt, 2015). Additionally, feedlot pens tend to be much larger (more than 100 calves per pen) which may negatively impact the external validity of these results.

For our question, the strength of evidence is weak overall because the reviewed papers are small experimental studies, and each have limitations to answering the presented question (Hagenmaier et al., 2018; and Rogers et al., 2016). Within the details of each study, we have provided a list of limitations either identified directly or limitations that result from a lack of detail in the manuscript. Biased results can confound information leading to incorrect findings therefore producers should bear in mind the limitations of each study when considering a change in their vaccine protocol based on this summary. 

*The two studies reviewed in this summary are randomised complete block designs, which places them within the classification randomised controlled trials which produce the strongest evidence of experimental studies.

## Summary of the evidence

**Rogers et al. (2016):** 

Population:	High-risk heifer calves from 274–295 kg; processed within 72 hours of arrival at the feedlot (the paper described the heifers as 'high-risk' and indicated they had one or more risk factors for BRD).
Sample size:	n = 5,179, (average 86 calves / pen; 15 pens / treatment).
Intervention details:	Treatments: Delayed vaccine 30 days (± 5 days) days on feed (DOF). Vaccine on arrival to feedlot and 30 DOF (± 5 days). Delayed vaccine plus DNA immunostimulant. Arrival vaccine plus DNA immunostimulant. Cattle received tilmicosin during processing for metaphylaxis but no post-metaphylactic interval was described. Vaccine: Modified-live infectious bovine rhinotracheitis virus (IBRV), parainfluenza-3 virus, bovine viral diarrhea virus (BVDV) types 1 and 2, and bovine respiratory syncytial virus vaccine (Pyramid 5, 2 mL subcutaneously in the right neck; Boehringer Ingelheim Vetmedica, Inc.).
Study design:	Randomised complete block design with a 2x2 treatment factorial.
Outcome studied:	Bovine respiratory disease (BRD) morbidity (cattle must have a rectal temperature of ≥40 °C and any one of the clinical signs of BRD [depression, lowered head carriage, nasal and / or ocular discharge, coughing, stiff gait, or depressed ruminal fossa]) OR if advanced signs of illness treatment was initiated even if rectal temperature did not meet cutoff – subjective assessment; blinded personnel. BRD treatments 1, 2, and 3 at 60 days on feed (DOF) – subjective assessment; blinded personnel. BRD 1 treatment rate at approximately 116 DOF (104–127 DOF). BRD retreatment number at approximately 116 DOF (104–127 DOF). BRD 1, 2, and 3 treatment-rate at closeout (average 209 days). BRD retreatment risk at closeout.
Main Findings (relevant to PICO question)	Delayed modified live vaccine (MLV) for viral respiratory pathogens statistically significantly reduced the number of calves requiring two treatments at 60 DOF (p = 0.04), at approximately 116 DOF (data not shown, p = 0.04), and at closeout (p = 0.04), and retreatment risk at approximately 116 DOF (p = 0.01) and retreatment risk at closeout (p = 0.01, 37.05% in delayed vs 43.97% in arrival). At 60 DOF, approximately 116 DOF, and closeout the percentage of calves that received one treatment was the same, statistically, between arrival versus delayed vaccine administration (p = 0.70, p = 0.78, and p = 0.82, respectively).
Limitations:	Model-adjusted means and confidence intervals for the overall effect of vaccine were not provided for all outcomes which limits the interpretation of the results as no magnitude of effect can be appreciated. Study included pregnant heifers (<90 days) and aborted with prostaglandin at processing, but there was no difference in the number among treatments, and heifers >90 days pregnant were excluded from the study. Pen size ranged from 79–98 heifers. DOF ranged from 196–221 days (209 days on average). BRD treatment details including antimicrobials administered and post-treatment intervals were not described.

**Hagenmaier et al. (2018):** 

Population:	High-risk beef heifers at 258 ± 12.7 kg (the paper described the heifers as 'high-risk' and indicated they had one or more risk factors for BRD).
Sample size:	n = 2,575, (average 86 heifers / pen, 10 pens / treatment).
Intervention details:	Treatments: Pyramid 5 and Presponse SQ during arrival: (PRE) (860 heifers in 10 pens). Nuplura PH and Titanium 5 during arrival processing: (TNA) (855 heifers in 10 pens). Nuplura PH during arrival with Titanium 5 delayed until 28 days after arrival processing: (TND) (860 heifers in 10 pens). No booster vaccines were given during this study. Cattle received timicosin as a metaphylactic treatment during arrival processing with a 3 day post-metaphylactic interval during which no animal could be diagnosed with bovine respiratory disease (BRD) and receive treatment. Vaccines: Pyramid 5: modified live infectious bovine rhinotracheitis, bovine viral diarrhea types I and II, parainfluenza 3, and respiratory syncytial virus vaccine (2 ml subcutaneously; Boehringer Ingelheim Vetmedica, Inc.). Presponse SQ: *Pasteurella multocida* bacterial extract and *Mannheimia haemolytica* toxoid (2 ml subcutaneously; Boehringer Ingelheim Vetmedica, Inc.). Titanium 5: modified live infectious bovine rhinotracheitis, bovine viral diarrhea virus types 1 and 2, bovine parainfluenza 3, and bovine respiratory syncytial virus vaccine (2 ml subcutaneously; Elanco Animal Health). Nuplura PH: toxoid and cellular-associated antigens of *Mannheimia haemolytica* cultures (2 ml subcutaneously; Elanco Animal Health).
Study design:	Randomised complete block design.
Outcome studied:	BRD treatment 1, 2, and 3 at reimplant (average 111 days) and closeout (average 219 days) subjective assessment; blinded evaluators. In order to be considered a BRD case and receive antibiotic treatment, heifers must have had a rectal temperature ≥40°C and at least one of the clinical signs indicative of BRD (depression / lethargy, incoordination, dypsnea / abnormal respiration [rate, character, etc.], sunken eyes / dehydration, nasal and / or ocular discharge, lowered head carriage, and / or depressed ruminal fossa). Non-febrile heifers with severe clinical presentation of BRD according to hospital personnel were also administered therapy without meeting the rectal temperature cutoff. A 3 day post-treatment interval was observed after BRD 1 (enrofloxacin), BRD 2 (florfenicol), and BRD 3 (oxytetracycline). Late day morbidities were treated with danofloxacin to remain within pre-slaughter withdrawal.
Main Findings (relevant to PICO question)	No statistically significant differences in morbidity assessments were found between vaccine treatments at reimplant or closeout. Note: only a P-value for PRE, TNA, and TND compared together was reported therefore pairwise comparisons for TNA vs TND were not available. At reimplant (average 111 days on feed [DOF]) BRD treatment 1, 2, and 3, were numerically greater in those receiving the vaccine at 28 DOF (TND) when compared to TNA. BRD 1: TND 14.57% vs TNA 12.39% BRD 2: TND 6.45% vs TNA 5.73% BRD 3: TND 3.10% vs TNA 2.39% At closeout (average 219 DOF) BRD treatment 1, 2, and 3, were also numerically greater in TND versus TNA. BRD 1: TND 15.77% vs TNA 13.24% BRD 2: TND 7.53% vs TNA 6.24% BRD 3: TND 3.62% vs TNA 3.10%
Limitations:	The treatment interventions TNA and TND were the only ones evaluated because the other intervention had variables inconsistent with the presented question. The timing of processing relative to feedlot arrival was unclear. Blocks were created over up to a 4 day period and a minimum of 24 hours rest was given to all cattle after the block was completed. All heifers were given a prostaglandin during arrival processing, but no pregnancy diagnosis was described. Standard errors for model-adjusted least square means for TNA and TND for individual outcomes were not reported. Pairwise comparisons and confidence intervals between TNA and TND were not reported which limits the ability to interpret the results relative to our question. Pen size ranged from 77–91 animals. Days on feed ranged from 189–238 days (average 219 days). Reimplant ranged from 110–113 days (average 111 days). Morbid cattle that were not well enough to thrive remained in the hospital pen while those who could thrive were returned to their home pens after treatment.

## Appraisal, application and reflection

Rogers et al. (2016) showed calves that received a delayed modified live vaccine (MLV) for viral respiratory pathogens were significantly less likely to require two treatments by 60 days on feed (DOF), by 116 DOF, and closeout. They also found a significant reduction in retreatment risk at 116 DOF and closeout in the delayed vaccine group (Rogers et al., 2016). In contrast, Hagenmaier et al. (2018) showed only a numerical reduction of bovine respiratory disease (BRD) treatments 1, 2, and 3 in calves receiving MLV for viral respiratory pathogens on arrival at reimplant (average 111 days) and closeout (average 219 days) (Hagenmaier et al., 2018) and did not statistically evaluate the pairwise comparison between the two treatment groups that were pertinent to our clinical question. These studies have inherent limitations as outlined below and differ in the number of animals evaluated, vaccine studied, timing of delayed vaccination and timing of health outcome measurement, health management, etc., which should be considered when evaluating the results.

Morbidity assessment is, ultimately, a subjective assessment although some case definitions include a rectal temperature cutoff. However, cattle temperatures are not taken unless respiratory illness signs are observed, so temperature data ultimately relies on that initial subjective assessment of clinical signs. The exact clinical signs used to identify sick cattle can and do vary between studies and operations. Additionally, the way an animal is deemed morbid varies between feedlot protocols and between the studies we evaluated. Our specific question was about cattle deemed morbid due to clinical signs and a rectal temperature of >40°C.  However, no studies were identified that met that exact question as both of the included studies allowed cattle with advanced clinical signs (per the blinded evaluator) to be treated even if they did not reach a rectal temperature of ≥40°C. Allowing treatment of animals that do not meet a rectal temperature threshold could introduce unwanted variation in the morbidity outcomes due to potentially more false positives. It also makes it more difficult to compare morbidity outcomes between studies as the variation related to what one person deems ‘severe’ or ‘advanced’ depends on training, experience, and the type of cattle they are evaluating. Due to this variation, animals that were deemed morbid in some studies may not have met criteria to be morbid in another study, so description of criteria for a morbid patient should be compared to a producer’s protocol when deciding whether a study provides good evidence to change practices. Additionally, it is important to also consider the way morbid cattle are handled including the antimicrobial therapies applied, any post-treatment intervals that are observed, whether cattle were hospitalised or returned to their home pen, etc., when evaluating studies. Hagenmaier et al. (2018) described these aspects of their health program but Rogers et al. (2016) did not fully describe treatment regimens which makes it difficult to compare between the health protocols, and thus health-related outcomes, of each study. Additionally, origins and prior health backgrounds of cattle represented within these studies differ as do the timing of arrival processing relative to feedlot arrival. This could be a strength of this summary due to our desire to broadly apply these findings to the diverse populations of cattle entering feedlots. However, it also introduces variability into the results of this Knowledge Summary as much of the prior health data and origin data are unknown. Another limitation is that the two studies considered in this Knowledge Summary only explored delayed vaccination in heifers, therefore the impact of gender, if any, cannot be evaluated (Hagenmaier et al., 2018; and Rogers et al., 2016). Finally, the fact that both studies had less animals per pen than would typically be found in a commercial setting could influence the external validity of the results as respiratory disease dynamics are often different when the pen size is smaller. Therefore, the answer to the presented question is, unsurprisingly, not consistent across the two studies we examined.

Even with the specific criteria for study selection that are outlined below, there is additional variability between studies that should be considered. Modified live respiratory vaccines for cattle are made by a variety of pharmaceutical companies and the differences between these vaccines can include: the adjuvant used, number of viruses covered by the vaccine, different pathogen loads, different virus types and strains included, etc. Each summary of the studies we evaluated includes a list of the current manufacturer, brand name, and the viruses included in the MLV respiratory vaccine administered. It is important to note that different vaccines and protocols were tested in the two studies we evaluated (Hagenmaier et al., 2018; and Rogers et al., 2016). Therefore, although both studies provide evidence for our question related to delayed vs on-arrival MLV use, they do not provide consistent evidence in terms of products used.

Producers should use the information provided in this summary to make vaccine protocol decisions in light of the limitations listed above. Since the evidence differed among studies, there was considerable variability between the management of the cattle between the two studies, and so few studies were identified, a definite answer cannot be given to the clinical question.

## Methodology Section

**Search Strategy:** 

Databases searched and dates covered:	CAB Abstracts via OVID 1910–2021 Week 50 Limit results to 2000–December 18, 2021 PubMed via NCBI Website Limit results to 2000–December 18, 2021 Limit results to English
Search strategy:	CAB Abstracts: ((exp cattle/ or (calf or calves or steer or steers or heifer or heifers or bull or bulls or bovine or bovines or cattle or youngstock or young-stock or (young adj2 stock)).mp.) and (exp vaccines/ or vaccin*.mp. or exp immunization/ or immuni*.mp.) and (delay or delays or delayed or arrive or arrives or arrived or arrival or postarrival or "post arrival" or post-arrival).mp. and (pneumon*.mp. or (respiratory adj1 disease*).ti,ab. or "respiratory diseases".sh. or (respiratory adj2 disease*).ti,ab. or ((shipping or undifferentiated) adj1 fever).ti,ab. or (BRD or BRDC).ti,ab. or (bovine adj1 respiratory adj1 disease*).ti,ab. or (bovine adj1 respiratory adj1 disease* adj1 complex).ti,ab. or (summer adj1 pneumon*).ti,ab. or (enzootic adj1 pneumon*).ti,ab. or pleuropneumon*.mp. or bronchopneumon*.mp. or (respiratory adj1 tract adj1 disease*).mp.)) PubMed: (("pneumonia"[MeSH Terms] OR "pneumonia"[Title/Abstract] OR "pneumoniae"[Title/Abstract] OR "pneumonias"[Title/Abstract] OR "respiratory diseases"[All Fields] OR "respiratory disease"[Title/Abstract] OR "shipping fever"[Title/Abstract] OR "undifferentiated fever"[Title/Abstract] OR "BRD"[Title/Abstract] OR "BRDC"[Title/Abstract] OR "bovine respiratory disease"[Title/Abstract] OR "bovine respiratory disease complex"[All Fields] OR "summer pneumonia"[Title/Abstract] OR "enzootic pneumonia"[Title/Abstract] OR ("pleuropneumonia"[MeSH Terms] OR "pleuropneumonia"[Title/Abstract] OR "pleuropneumonias"[Title/Abstract] OR "pleuropneumoniae"[Title/Abstract]) OR ("bronchopneumonia"[MeSH Terms] OR "bronchopneumonia"[Title/Abstract] OR "bronchopneumonias"[Title/Abstract] OR "bronchopneumoniae"[Title/Abstract]) OR "respiratory tract disease"[Title/Abstract] OR "respiratory tract diseases"[All Fields]) AND ("calf"[Title/Abstract] OR "calves"[Title/Abstract] OR "steer"[Title/Abstract] OR "steers"[Title/Abstract] OR "heifer"[Title/Abstract] OR "heifers"[Title/Abstract] OR "bull"[Title/Abstract] OR "bulls"[Title/Abstract] OR "bovine"[Title/Abstract] OR "bovines"[Title/Abstract] OR "cattle"[Title/Abstract] OR "cattle"[MeSH Terms] OR "youngstock"[Title/Abstract] OR "young-stock"[Title/Abstract] OR "young-stock"[Title/Abstract]) AND ("immunization"[MeSH Terms] OR "immunization"[Title/Abstract] OR "immunisation"[Title/Abstract] OR "immunizations"[Title/Abstract] OR "immunisations"[Title/Abstract] OR "immunize"[Title/Abstract] OR "immunise"[Title/Abstract] OR "immunized"[Title/Abstract] OR "immunised"[Title/Abstract] OR "vaccination"[MeSH Terms] OR "vaccine"[Title/Abstract] OR "vaccines"[Title/Abstract] OR "vaccination"[Title/Abstract] OR "vaccinating"[Title/Abstract] OR "vaccinated"[Title/Abstract]) AND ("delay"[Title/Abstract] OR "delayed"[Title/Abstract] OR "delays"[Title/Abstract] OR "post-arrival"[Title/Abstract] OR "post-arrival"[Title/Abstract] OR "postarrival"[Title/Abstract] OR "arrive"[Title/Abstract] OR "arrival"[Title/Abstract] OR "arrives"[Title/Abstract] OR "arrived"[Title/Abstract])
Dates searches performed:	18 Dec 2021

**Exclusion / Inclusion Criteria:** 

Exclusion:	Publication date prior to 2000, not a research trial, does not compare arrival versus delayed vaccine administration groups, does not evaluate the same vaccine given on arrival versus delayed administration, does not evaluate the bovine species, or calves not evaluated in a feedlot.
Inclusion:	BRD morbidity assessment, comparison of effects delayed modified live vaccine administration and administration of vaccine on arrival to the feedlot, and respiratory vaccine used when evaluating vaccine timing.

**Search Outcome:** 

Database	Number of results	Excluded – Not a research trial	Excluded – Not an MLV respiratory vaccine study in calves	Excluded – Does not compare arrival versus delayed (14–30 days) administration of the same MLV vaccine	Excluded – Calves not evaluated or administered treatment in the feedlot	Total relevant papers
CAB Abstracts	115	21	47	40	6	1
PubMed	50	7	16	24	3	0
Hand Search of reference lists						1
Total relevant papers when duplicates removed						2

## Conflict of Interest

Moberly serves on the CABI Publishing North American Library Advisory Board and the VetStream Academic Advisory Board.

Capik has previously collaborated with a co-author (M. Theurer) on the Hagenmaier et al. (2018) paper.

## Disclaimer

Knowledge Summaries are a peer-reviewed article type which aims to answer a clinical question based on the best available current evidence. It does not override the responsibility of the practitioner. Informed decisions should be made by considering such factors as individual clinical expertise and judgement along with patient’s circumstances and owners’ values. Knowledge Summaries are a resource to help inform and any opinions expressed within the Knowledge Summaries are the author’s own and do not necessarily reflect the view of the RCVS Knowledge. Authors are responsible for the accuracy of the content. While the Editor and Publisher believe that all content herein are in accord with current recommendations and practice at the time of publication, they accept no legal responsibility for any errors or omissions, and make no warranty, express or implied, with respect to material contained within. For further information please refer to our Terms of Use.
